# Lethal and Sublethal Effects of Cyantraniliprole on the Biology and Metabolic Enzyme Activities of Two Lepidopteran Pests, *Spodoptera littoralis* and *Agrotis ipsilon,* and A Generalist Predator, *Chrysoperla carnea* (Neuroptera: Chrysopidae)

**DOI:** 10.3390/insects15060450

**Published:** 2024-06-13

**Authors:** Mona Awad, Ahmed H. El Kenawy, Nawal AbdulAziz Alfuhaid, El-Desoky S. Ibrahim, Júlia Katalin Jósvai, Adrien Fónagy, Moataz A. M. Moustafa

**Affiliations:** 1 Department of Economic Entomology and Pesticides, Faculty of Agriculture, Cairo University, Giza 12613, Egypt; mona_sonson@agr.cu.edu.eg (M.A.); aldosokyibrahim@yahoo.com (E.-D.S.I.); 2 Biological Control Department, Agricultural Research Centre, Giza 12619, Egypt; ahmed.hamed.elkenawy@gmail.com; 3 Department of Biology, College of Science and Humanities, Prince Sattam Bin Abdulaziz University, Al-Kharj 11942, Saudi Arabia; n.alfuhaid@psau.edu.sa; 4 Department of Chemical Ecology, Plant Protection Institute, HUN-REN Centre for Agricultural Research, 1022 Budapest, Hungary; josvai.julia@atk.hu

**Keywords:** diamide insecticide, noctuid moths, chrysopidae, selectivity, detoxification enzymes, generalist biological control agent

## Abstract

**Simple Summary:**

This study discusses the effects of cyantraniliprole, a new insecticide, on two lepidopteran pests and a biological control agent. Cyantraniliprole showed higher toxicity to one pest species compared to the other, prolonged developmental stages, and increased detoxification activity in both pest species. It also had a negative but slight impact on the biological control agent at the recommended concentrations. The study suggests that cyantraniliprole can effectively control the pests while being compatible with the control agent, but further field studies are needed for validation.

**Abstract:**

Cyantraniliprole is a novel anthranilic diamide insecticide registered for controlling chewing and sucking insect pests. Here, the lethal and sublethal effects of this insecticide on two destructive lepidopteran pests, *Spodoptera littoralis* Boisduval and *Agrotis ipsilon* Hufnagel, were evaluated. Because the effects of novel insecticides on beneficial and non-target arthropods must be considered, the impact of cyantraniliprole on a generalist biological control agent, *Chrysoperla carnea* [Stephens 1836], were also examined. Overall, our study revealed that cyantraniliprole was more toxic to *A. ipsilon* than to *S. littoralis*. Moreover, the LC_15_ and LC_50_ of the insecticide significantly prolonged the duration of the larval and pupal stages and induced enzymatic detoxification activity in both species. Treatment of the second-instar larvae of *C. carnea* with the recommended concentration of cyantraniliprole (0.75 mg/L) doubled the mortality rates and resulted in a slight negative effect on the biology and detoxification enzymes of *C. carnea*. Our results indicate that both sublethal and lethal concentrations of cyantraniliprole can successfully suppress *S. littoralis* and *A. ipsilon* populations. They also suggest that *C. carnea*, as a generalist predator, is compatible with cyantraniliprole under the modelled realistic field conditions. In future investigations, insights into the effects of cyantraniliprole on *S. littoralis*, *A. ipsilon*, and *C. carnea* under field conditions will be required to appropriately validate our results.

## 1. Introduction

Economic losses in agriculture due to insect pests often necessitate the use of chemical pesticides [1], which have historically been the primary method for controlling pest populations. In recent decades, alternative control methods, such as biological control, have become more appealing [2]. Given that one primary focus of integrated pest management (IPM) is the use of non-chemical control measures, the applicability of biological control methods measures is under constant consideration [3]. However, natural enemies are not always successful in pest management, and more immediate corrective interventions to suppress the growth of pest populations are often needed [3,4]. One limitation of relying on chemical control methods is that broad-spectrum insecticides can interfere with natural biological control [5]. Therefore, it is crucial to assess the impact of insecticides on biological control agents when developing effective pest control strategies [6,7].

Noctuidae is the most challenging family in the Noctuoidea superfamily [8]. It consists of about 1150 genera comprised of more than 12,000 diverse and ecologically successful species [9,10]. Noctuid moths are the most important agricultural pests globally and include species capable of adapting to new climatic or ecological situations, such as cotton leafworm, *Spodoptera littoralis* [Boisduval, 1833], and black cutworm, *Agrotis ipsilon* [Hufnagel, 1766] [11]. Unfortunately, both insects have developed resistance to conventional insecticides, including organophosphates, carbamates, and pyrethroids [12,13,14], as well as to several newer insecticides [15,16,17]. *S. littoralis* is a highly polyphagous and destructive pest that attacks a wide range of economically important crops throughout the world and causes yield losses of up to 50%, typically due to larval foraging activity [18,19]. *A. ipsilon* is also a major polyphagous pest that damages more than 30 important crops worldwide [20]. The larvae of *A. ipsilon* can consume more than 400 cm^2^ of foliage during their development [21,22].

As a potential predator, the green lacewing, *Chrysoperla carnea* [Stephens, 1836] (Neuroptera: Chrysopidae), is a polyphagous foliage-dwelling predator that has been promoted as a biological agent for a wide range of pest species, including aphids, lepidopteran eggs and neonates, whiteflies, leafhoppers, scales, mites, and other soft-bodied insects [23,24,25]. Several studies have been performed on the predation capacity of *C. carnea* on lepidopteran prey [26,27,28], including eggs and neonate larvae of *S. littoralis* [29] and cotton bollworm, *Helicoverpa armigera* [Hübner, 1808] [30]. Although green lacewings are found in a wide range of agroecosystems [25], they are mainly utilized to control greenhouse and outdoor crop pests through augmentative releases [31,32]. The excessive and indiscriminate use of pesticides [33] has negatively impacted field populations of *C. carnea* [34,35] and necessitated the use of more selective insecticides.

Diamide insecticides have emerged as the most recent and successful chemistries for controlling both sap-feeding insect pests and chewing pests [36]. Several studies have shown that this group has a high insecticidal activity on different lepidopteran species such as tobacco cutworm, *Spodoptera litura* [Fabricius, 1775] [37], *Spodoptera exigua* (Hübner, 1808) [38], *S. littoralis* [39], *A. ipsilon* [13,22], and *Mamestra brassicae* [Linnaeus, 1758] [40]. Unfortunately, diamide insecticides can also harm natural arthropod enemies in agricultural systems, thus triggering secondary pest outbreaks [41].

Cyantraniliprole is a second-generation diamide insecticide that is widely used to control various lepidopteran, coleopteran, dipteran, and hemipteran pests [42,43]. As it has systemic and translaminar behavior, it can be employed in soil or as a foliar application for pest management [44]. As a highly potent agonist of insect ryanodine receptors, cyantraniliprole activates the ryanodine receptor channels [45] that disrupt calcium balance.

Generally, insecticides have varying effects on insect defense enzyme systems, which are usually associated with insect death or the development of resistance. Several studies have shown that insects protect themselves against insecticides by increasing the activity of detoxifying enzymes such as cytochrome P450 monooxygenases (P-450s), esterases, and glutathione S-transferases (GSTs) [39]. Additionally, the insecticide breakdown often exposes insects to sublethal concentrations following application in crops, which impacts various biological and biochemical parameters [46]. So, determining the sublethal effects of insecticides on insects is crucial for understanding their impact on agroecosystems and prolonging the long-term efficiency of these insecticides in insect management. Therefore, this study aims to assess the effect of cyantraniliprole on two lepidopteran pests, *S. littoralis* and *A. ipsilon*, and on *C. carnea* as a potential predator of the two species. This study will hopefully allow for a better understanding of pest and predator interactions and help develop a more effective IPM program.

## 2. Materials and Methods

### 2.1. Insect Cultures

#### 2.1.1. Target Pests

Cultures of *S. littoralis* and *A. ipsilon* were obtained from the Department of Entomology at the Faculty of Agriculture, Cairo University, Egypt. *S. littoralis* was maintained in the laboratory at 25 ± 2 °C and 55 ± 5% RH [47,48,49], while *A. ipsilon* was maintained at 27 ± 2 °C and 55 ± 5% RH [48,49]. Sexed pupae of both insects were kept covered in glass jars lined with paper towels until adult emergence. The incipient adult moths (seven males and five females) were transferred to a larger jar draped with cotton wool soaked in a 10% sugar solution as dietary supplement [46]. The deposited eggs were collected daily and transferred to new jars for hatching [14]. Neonates were fed castor oil leaves, and a colony of mass-reared larvae was maintained as described above. Experimental treatments were conducted using the second-instar larvae of *S. littoralis* and *A. ipsilon*.

#### 2.1.2. Non-Target Pest

*C. carnea* larvae were obtained from the Biological Control Laboratory in Aswan Governorate, Egypt. Larval cultures were reared on UV-sterilized eggs of *Ephestia kuehniella* [Zeller, 1879] that were stored at 2–4 °C. Adults were maintained on an artificial diet consisting of honey, yeast, and water (7:4:4, respectively) in sterile plastic containers (17 × 25 cm) covered with muslin cloth in insect rearing room at 25 °C and 60% RH and 14:10 h- dark–light intervals. The colony was maintained for two generations without an infusion of wild stock before experiments on the second-instar larvae began.

### 2.2. Insecticide

The commercial formulation of cyantraniliprole *Benevia 100 OD* (FMC Company, Philadelphia, PA, USA) was used in this study with a field rate of application of 75 mL/100 L water.

### 2.3. Toxicity of Cyantraniliprole to Target Species

Cyantraniliprole toxicity bioassays on newly ecdysed second-instar larvae of *S. littoralis* and *A. ipsilon* were performed as described by [39] and [22]. Insecticide dosages were optimized in preliminary studies. Five concentrations (5, 2.5, 1.25, 0.125, and 0.0125 mg/L) of cyantraniliprole were used to determine LC_15_ and LC_50_, and experiments were repeated twice to confirm the results. The leaf-dipping technique was used according to [50] as follows: for experiments, castor leaves were dipped in each concentration for 20 s and then allowed to dry in the air for 30 min. Five replicates with ten larvae each were used for each concentration. Leaves dipped in water were used in the control group. The larvae were allowed to feed on treated leaves for 24 h, and the surviving larvae were then transferred to a clean dry container containing fresh untreated leaves [39,50]. Larval mortality was recorded daily for four consecutive days post-treatment, and the lethal and sublethal concentrations were calculated [14] and corrected using Abbott’s formula [51].

### 2.4. Sublethal and Lethal Effects of Cyantraniliprole on Target Species

The impact of the cyantraniliprole LC_15_ and LC_50_ on *S. littoralis* and *A. ipsilon* development was assessed. Three replicates with 50 larvae each were used for each concentration/control (n = 150). Ninety-six hours after treatment, the surviving larvae were kept individually in a small, dry, clean cup with fresh, untreated castor leaves [14]. Developmental changes, i.e., larval and pupal durations (days), pupation percentage, pupal weight [g], adult emergence percentage, and sex ratio of *S. littoralis* and *A. ipsilon*, were recorded daily [50]. Additionally, fecundity and hatchability percentages were assessed using three replicates, each of five females and seven males [52].

### 2.5. Sublethal Effects of Cyantraniliprole on C. carnea

Toxicity bioassays were conducted on the second-instar larvae of *C. carnea* in a controlled chamber using two methods under standardized environmental conditions. Three concentrations of cyantraniliprole were used: the recommended concentration (0.75 mg/L), half the recommended concentration (0.37 mg/L), and a quarter of the recommended concentration (0.19 mg/L). To assess the acute toxicity of cyantraniliprole to *C. carnea* larvae, the larval mortality was recorded 24 h post-treatment and then for 120 h. Two insecticide contamination methods were used:

1. The direct contact method: IOBC standard guidelines for *C. carnea* [53] were followed with some modifications. To estimate the residual effects of cyantraniliprole on *C. carnea* larvae, filter paper was dipped for 5 s in each concentration. For the control group, the filter paper was dipped in water. The treated and untreated paper was then allowed to dry in the air. For each treatment, five replicates with 10 larvae each were used. The larvae were placed on the contaminated filter paper for 24 h, after which they were transferred to individual Petri dishes (5 cm) to avoid cannibalism and provided with *E. kuehniella* eggs every two days.
2. The insecticide-treated host method (feeding method): *E. kuehniella* egg cards [1 × 1 cm] were dipped for 5 s in cyantraniliprole concentrations, and each card was then placed in a 5 cm Petri dish with a single second-instar larva of *C. carnea* for 24 h before being replaced by an untreated one.

### 2.6. Sublethal Effects on C. carnea

The effect of field exposure to cyantraniliprole on the larval and pupal duration (days), pupation percentage, and emergence percentage was assessed. For each treatment, five replicates of second-instar larvae, each comprising 10 individuals, were utilized. The following formulas were used to calculate pupation and emergence percentages: pupation (%) = 100 × (total number of pupae/total number of larvae), adult emergence (%) = 100 × (total number of adults/total number of pupae).

### 2.7. Biochemical Assays

#### 2.7.1. Sample Preparation

Second-instar larvae of the three insects were exposed to LC_15_ and LC_50_ of cyantraniliprole, as described above. A total of 50 mg fresh body weight of the surviving larvae were then used to measure the activities of detoxifying enzyme at 24, 48, 72, and 96 h post-treatment in *S. littoralis* and *A. ipsilon* and 120 h post-treatment in *C. carnea*. Five separate replicates were used for each analysis. The larvae were homogenized in 0.1 M phosphate buffer, pH 7.0 for carboxylesterase (CarE), or pH 6.5 for glutathione S-transferase (GST). The homogenates were centrifuged at 7000 rpm for 15 min, and the supernatants were used to determine enzyme activity and protein content, as described below.

#### 2.7.2. CarE Assay

Alpha (*α*)- and beta (*β*)-esterase activities were measured as described by [54] and [40]. The homogenate was incubated for 15 min at 25 °C with alpha or beta-naphthyl acetate as the substrate. A mixture of Fast Blue B and sodium dodecyl sulfate was added to stop the reaction. The optical density was measured at 550 nm for *α*- esterase and 600 nm for *β*- esterase using a Jenway Spectrophotometer-7205UV/Vis, Dunmow, Essex, UK.

#### 2.7.3. GST Assay

GST activity was determined as described by [55] and [40]. The assay mixture contained the sample enzyme solution with CDNB (1-chloro-2, 4-dinitrobenzene) as the substrate and GSH as a reagent. The optical density was recorded at 340 nm for 3 min with readings taken at 1-min intervals, using a Jenway Spectrophotometer-7205 UV/Vis., UK.

#### 2.7.4. Protein Content

Coomassie brilliant blue assay [56] was used to determine the protein concentration.

### 2.8. Data Analysis

Four days post-exposure to cyantraniliprole, LC_15_ and LC_50_ to the second-instar larvae of *S. littoralis* and *A. ipsilon* were estimated using Probit analysis (EPA Probit analysis program, V. 1.5) [57]. All the biological and biochemical data were coded and entered using the statistical package SPSS V.22. Data were tested for satisfying assumptions of parametric tests, and continuous variables were subjected to the Shapiro–Wilk and Kolmogorov–Smirnov test for normality. Probability and percentile data were standardized for normality using Arcsine Square Root. Data were presented as mean and standard deviation. ANOVA was done for experimental groups using three replicates at least for each group, while post-hoc analysis was done using Tukey’s pairwise comparison. *p*-value was considered significant at <0.05. Chi (ꭓ^2^) was used for comparing the observed and expected frequencies of sex ratio using MiniTab (V. 14). Finally, data were visualized using R studio (V. 2022.02.4).

## 3. Results

### 3.1. Toxicity of Cyantraniliprole to S. littoralis and A. ipsilon

As shown in Table 1, the results of cyantraniliprole toxicity to the second-instar larvae of both insects revealed that *S. littoralis* larvae were more tolerant than *A. ipsilon*. The cyantraniliprole LC_15_ values were 1.95 and 0.019 mg/L, whereas the LC_50_ values were 8.17 and 0.33 mg/L to *S. littoralis* and *A. ipsilon,* respectively.

### 3.2. Toxicity of Cyantraniliprole to C. carnea

**Figure 1:**
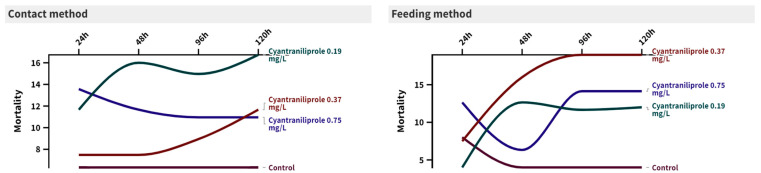
Mortality percentages of *C. carnea* at 24, 48, 96, and 120 h post-treatment with three concentrations (0.19, 0.37, and 0.75 mg/L) of cyantraniliprole using two different methods of exposure.

The toxicity of three concentrations of cyantraniliprole to the second-instar larvae of *C. carnea* is presented in Figure 1. It can be observed that the interactions between time and treatment were not significant for either the direct contact method or the feeding method. The recommended concentration (0.75 mg/L) of cyantraniliprole showed the highest mortality % after 24 h in both contact and feeding methods (24 ± 13.56 (F = 2.29, *p* = 0.117) and 10 ± 12.64 (F = 0.63, *p* = 0.606), respectively), compared to the control (10 ± 6.32 and 6 ± 8, respectively). After 120 h, the mortality % insignificantly increased, compared to the control, for both methods (30 ± 10.95 (F = 1.88, *p* = 0.174) and 30 ± 14.14 (F = 1.45, *p* = 0.265), respectively).

### 3.3. Lethal and Sublethal Effects of Cyantraniliprole

#### 3.3.1. On *S. littoralis* and *A. ipsilon*

The effects of cyantraniliprole on the development of *S. littoralis* and *A. ipsilon* are presented in Table 2 and Table 3. Treating the freshly molted second-instar larvae of both insects with LC_15_ and LC_50_ of cyantraniliprole resulted in the significant prolongation of larval (F = 106.98, *p* = 0.000 and F = 11.15, *p* = 0.000, respectively) and pupal (F = 48.40, *p* = 0.000 and F = 91.91, *p* = 0.000, respectively) durations (Table 2). Nevertheless, no significant differences were observed (Table 2) in the pupation rate (F = 4.45, *p* = 0.065 and F = 0.65, *p* = 0.556, respectively), emergence percentages (F = 3.0, *p* = 0.125 and F = 0.25, *p* = 0.788, respectively), or sex ratio (Table 3) of the emerged adults for both S. *littoralis* and *A. ipsilon*, respectively. Similarly, no significant differences (F = 1.26, *p* = 0.348 and F = 0.68, *p* = 0.543, respectively) were observed in fecundity (eggs laid per female) for both S. *littoralis* and *A. ipsilon*, respectively (Table 2).

As for the hatchability rate, it significantly decreased in both species after treatment with the LC_50_ of cyantraniliprole. For *S. littoralis*, cyantraniliprole treatments also caused a significant reduction in pupal weights in both males (F = 19.59, *p* = 0.000) and females (F = 29.45, *p* = 0.000) compared to the control. For *A. ipsilon*, a significant difference in pupal weight was only observed in males (F = 3.58, *p* = 0.032), while in females (F = 1.33, *p* = 0.270), there was no significant difference compared to the control treatment (Table 2).

#### 3.3.2. On *C. carnea*

The effects of cyantraniliprole on *C. carnea* development are presented in Table 4. Regarding the effect on larval duration, pupation rate, and pupal duration, no two-way interaction was detected between the tested concentrations and the contamination methods (two-way ANOVA, *p* > 0.05). Additionally, cyantraniliprole did not affect larval duration, pupation rate, or pupal duration at any concentration. Although cyantraniliprole decreased the emergence ratio at all concentrations, compared to the control, this reduction was statistically significant only at 0.37 and 0.75 mg/L (F = 25.12, *p* = 0.011) in the contact method. However, at a concentration of 0.75 mg/L cyantraniliprole significantly reduced the emergence ratio by 1.6-fold (F = 4.26, *p* = 0.022) in the feeding method, compared to the control.

### 3.4. Effect of Cyantraniliprole on Detoxifying Enzymes

#### 3.4.1. *S. littoralis* and *A. ipsilon*

**Figure 2:**
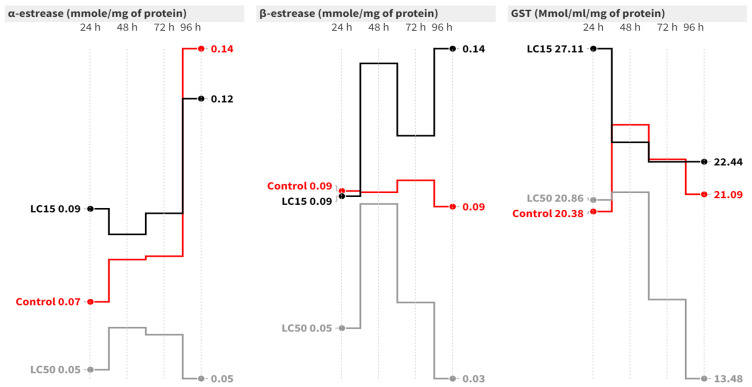
Mean of the detoxification enzyme activity of carboxylesterase (*α*- and *β*-esterase) and GST at 24, 48, 72, and 96 h post-treatment of *S. littoralis* second-instar larvae with LC_15_ and LC_50_ of cyantraniliprole.

In the case of *S. littoralis*, the elapsed time had no significant effect on the α-esterase activity at the LC_50_ level (Figure 2). At 24 and 96 h post-contamination with LC_50_, α-esterase activity significantly decreased by 2.25- [F = 11.32, *p* = 0.009] and 3.07-fold [F = 8.35, *p* = 0.018], respectively, compared to the control. Concerning the *β*-esterase assay, both the elapsed time and treatment had significant effects. The LC_15_ caused a significant increase in *β*-esterase activity at 48 and 96 h post-contamination compared to the control group [Figure 2]. On the other hand, the LC_50_ significantly increased *β*-esterase activity at all intervals except after 96 h, where the activity dropped to 0.03 mmol/mg of protein [F = 33.76, *p* = 0.001] compared to the control [Figure 2].

Concerning GST activity in *S. littoralis* larvae, the elapsed time did not significantly change the GST activity in the control or the LC_15_ groups. However, treatment with LC_50_ caused a significant reduction in GST activity at 96 h post-contamination (*F* = 23.64, *p* = 0.001) compared to the control (Figure 2).

**Figure 3:**
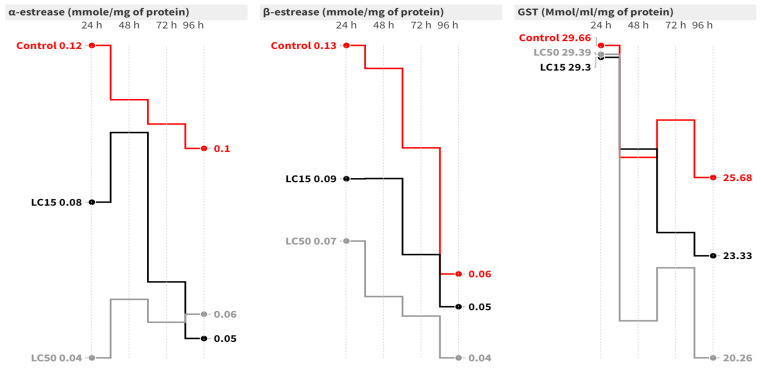
Mean of the detoxification enzyme activity of carboxylesterase (*α*- and *β*-esterase) and GST at 24, 48, 72, and 96 h post-treatment of *A. ipsilon* second-instar larvae with LC_15_ and LC_50_ of cyantraniliprole.

In the case of *A. ipsilon,* at 96 h post-contamination, the LC_15_ of cyantraniliprole caused a significant inhibition of 2.25-fold [F = 9.20, *p* = 0.015] in the *α*-esterase activity compared to the control group [Figure 3]. On the other hand, the LC_50_ of cyantraniliprole significantly decreased *α*-esterase activity at 24 [F = 7.80, *p* = 0.021], 48 [F = 17.49, *p* = 0.003], 72 (F = 6.44, *p* = 0.032) and 96 h (*F* = 9.20, *p* = 0.015) post-contamination. Concerning *β*-esterase, the highest activity was recorded in the control group, while significantly lower activity was observed in the LC_15_ and LC_50_ groups. The LC_15_ caused a significant inhibition in *β*-esterase activity at 48 h (F = 25.34, *p* = 0.001) post-contamination, while the LC_50_ significantly decreased the activity at 24 (F = 7.63, *p* = 0.022), 48 (F = 25.34, *p* = 0.001), 72 (F = 11.40, *p* = 0.009), and 96 h (F = 1.73, *p* = 0.254) post-contamination = compared to the untreated group (Figure 3). Concerning GST activity, it insignificantly declined at all intervals in all groups compared to the control group (Figure 3).

#### 3.4.2. *C. carnea*

**Figure 4:**
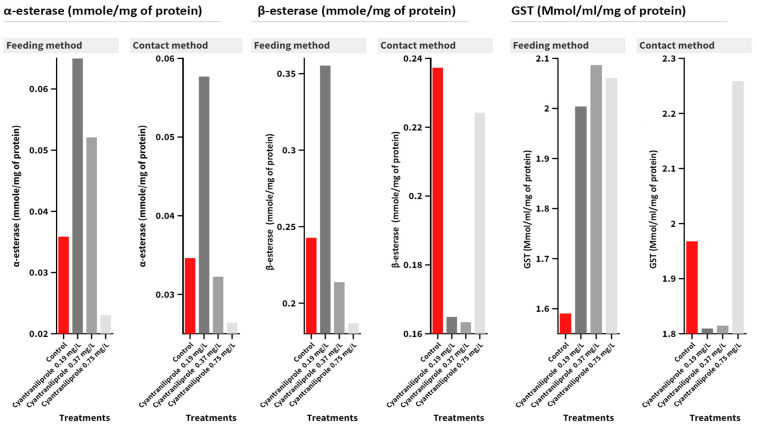
Mean of the detoxification enzyme activity of carboxylesterase (*α*- and *β*-esterase) and GST at 96 h post-treatment of *C. carnea* second-instar larvae with the recommended (0.75 mg/L), half recommended (0.37 mg/L), and quarter recommended (0.19 mg/L) dosages of cyantraniliprole.

In the case of α-esterase activity, no significant interaction was detected between the treatment and administration method (Figure 4). Cyantraniliprole treatments insignificantly affected the activity, while no effect was recorded in the feeding method (F = 3.56, *p* = 0.067) or the contact one (F = 6.13, *p* = 0.018). As for *β*-esterase activity, the interaction between treatments and contamination methods was insignificant. Except in 0.19 mg/L-treated larvae in the feeding contamination method (F = 12.94, *p* = 0.002), there were significant differences when compared with the control. As for GST activity, in the feeding method, there was a significant difference (F = 3.92, *p* = 0.054), while in the contact method, there were no significant differences in GST activity (F = 0.89, *p* = 0.487) compared to the control treatment (Figure 4).

## 4. Discussion

The management of arthropod pests in vegetables and high-value crops relies heavily on chemical control. However, there are many insecticide application options to choose from, and each differs in its effectiveness, costs, and risks to the environment and human health [58,59,60,61]. Therefore, it is necessary to make well-founded decisions that consider how pesticides impact all aspects of pest management including natural enemies, crop production, economic outcomes, and resistance [16,62]. In this study, we assessed the effects of cyantraniliprole as a novel insecticide on the potential management of two destructive lepidopteran pests, *S. littoralis* and *A. ipsilon.* Additionally, we assessed the adverse effect of this insecticide on the biological control agent *C. carnea.*

Cyantraniliprole exhibited insecticidal activity against the second-instar larvae of both *S. littoralis* and *A. ipsilon.* The LC_50_ to *S. littoralis* was significantly higher (~24.7-fold) than that to *A. ipsilon*. This indicates that *A. ipsilon* is more susceptible to cyantraniliprole. This is consistent with [13], who found, using an artificial diet, that cyantraniliprole caused high mortality in the fourth-instar *A. ipsilon* larvae (LC_50_ of 0.354 μg g^−1^). In addition to mortality, sublethal effects on larval development due to perturbations in the development of neural tissues by neurotoxic substances [35] may play a key role in managing insect pests [63]. In the current study, the larval and pupal durations of *S. littoralis* and *A. ipsilon* were prolonged due to exposure to cyantraniliprole. This could indicate that cyantraniliprole-treated larvae invest more resources in detoxification rather than development, and as a result, development is slowed relative to the control group [64]. Accordingly, pupal weight significantly decreased in all treatments, compared to the control groups. Similarly, the hatching rate decreased in both species as the cyantraniliprole LC_50_ significantly reduced emergence ~1.2 and 1.17 times in *S. littoralis* and *A. ipsilon*, respectively. Additionally, LC_50_ of cyantraniliprole insignificantly reduced the number of eggs laid per family ~1.4 and 1.2 times in both species, respectively, as in several studies that reported that the average number of eggs laid by adult females decreased after treatment with anthranilic diamide [13,38,65,66].

Insects in the natural environment are exposed to lethal and sublethal concentrations of insecticides [67], which may affect diverse enzymatic activities [68]. Generally, detoxification enzyme activity, such as CarEs and GSTs, can be useful for monitoring insecticide resistance development [69,70]. In addition, these enzymes are one of the adaptation mechanisms that enhance the insect’s metabolic capacity to counteract pesticides [71]. Thus, the competence of insects to detoxify is reflected by the reaction of detoxification enzymes to insecticides [17]. The exposure to lethal and sublethal concentrations (LC_50_ and LC_15_) of cyantraniliprole induced the activity of detoxification enzymes in both *S. littoralis* and *A. ipsilon* compared to the control group. In addition, medium to high levels of diamide resistance could be mediated by target site mutations and enhanced detoxification triggered by up-regulating the expression of genes encoding detoxification enzymes [72]. As revealed by our results, α-esterase activity significantly increased 1.3-fold after the *S. littoralis* second-instar larvae were treated with the LC_15_. Overall, this suggests that α-esterase is a key enzyme in *S. littoralis* detoxification. Likewise, [68] showed that a sublethal concentration of cyantraniliprole significantly increased CarE activity in the small brown planthopper *Laodelphax striatellus* [Fallén, 1826]. The activities of *S. littoralis β*-esterase and GST, however, decreased after treatment with the cyantraniliprole LC_50_. This decrease in detoxification enzyme activity is typically considered a positive marker of delayed resistance to toxic compounds [73]. Comparable results were observed in the activities of *A. ipsilon* CarE and GST following exposure to sublethal dosages of cyantraniliprole.

As new insecticides are registered, their effects on the arthropod community must be evaluated to ensure the important ecosystem services provided by predators in the farming system. Thus, the use of selective insecticides is crucial for maintaining the integral role of biological control in the food web [74,75]. Indiscriminate pesticide usage in agricultural fields can disrupt beneficial insect activities by reducing their overall abundance and species composition [76]. For example, some insecticides can block physiological or biochemical processes that affect the survival, growth, development, reproduction, and/or behavior of some natural enemy species [76,77]. Our study evaluated the effects of cyantraniliprole on *C. carnea*. Although cyantraniliprole had no significant effects on *C. carnea* larval mortality (two-fold increase with 0.75 mg/L at 24 h), the recommended dose of the insecticide prolonged larval duration and significantly reduced *C. carnea* emergence (Table 4). However, a potential effect of several conventional chemical insecticides has been observed on non-target organisms including *Chrysoperla zastrowi* (Sillemi) [78], *Chrysoperla nipponensis* (Steinmann, 1964) [79], and *C. carnea* [80]. Evaluating lifetable parameters can also provide insights into the response of natural enemies to selective insecticides from a population viewpoint [81]. Although cyantraniliprole at concentrations of 0.37 and 0.72 mg/L decreased the *C. carnea* emergence ratio relative to the control group, no significant differences were observed in larval duration, pupal duration, or pupation rate compared to the control. In addition, there was no significant interaction between the treatment and contamination methods when detoxification enzyme activities were assayed. However, the 0.19 mg/L cyantraniliprole concentration significantly increased α-esterase activity compared to the control and the two other concentrations evaluated. On the other hand, significant effects on *C. carnea* GST activity were observed only after 96 h, regardless of treatment or contamination method at LC_50_ value. Generally, arthropods rely heavily on detoxification enzymes for endogenous insecticide production. These enzymes can remove toxins and detoxify insecticides [82]. Therefore, several studies elucidated the role of these enzymes on lepidopteran insect pests [17,22,39,62], which suggests the possibility of discovering new insecticidals that act by interfering with metabolic-mediated enzymes in insects [83].

## 5. Conclusions

Overall, our study shows that cyantraniliprole is more toxic to *A. ipsilon* than to *S. littoralis.* Moreover, the LC_15_ and LC_50_ can significantly prolong both larval and pupal duration, which could help in the management of both pest populations. On the other hand, the use of selective insecticides in conjunction with biocontrol agents will increase biological efficacy and reduce the environmental consequences of incorporating insecticides in IPMs. Our data suggest that the predator *C. carnea* is compatible with cyantraniliprole under the modeled realistic field conditions. In future investigations, insights into the effects of cyantraniliprole on *A. ipsilon*, *S. littoralis, and C. carnea* under field conditions will be required to appropriately validate our results.

## Figures and Tables

**Table 1:** Toxicity of cyantraniliprole to the second-instar larvae of *S. littoralis* and *A. ipsilon*.

Insect Species	LC_15_ (mg/L) (95% Confidence Limits)	LC_50_ (mg/L) (95% Confidence Limits)	Slope ± SE	ꭓ^2^ [df]
*S. littoralis*	1.958 (0.659–3.404)	8.175 (5.290–11.362)	1.66 ± 0.30	0.52 [2]
*A. ipsilon*	0.019 (0.004–0.051)	0.338 (0.160–0.699)	0.83 ± 0.14	0.44 [2]

**Table 2:** Effects of cyantraniliprole on the development, fecundity, and fertility of *S. littoralis* and *A. ipsilon*.

	Sex		Control	LC_15_	LC_50_
Larval duration ^1^		*S. littoralis*	15.96 ± 1.46 ^c^	18.6 ± 1.01 ^b^	19.46 ± 1.93 ^a^
		*A. ipsilon*	19.21 ± 2.3 ^b^	19.79 ± 2.31 ^b^	21.38 ± 3.23 ^a^
Pupation %		*S. littoralis*	95.1 ± 3.5 ^a^	95.53 ± 4.15 ^a^	86.03 ± 3.04 ^a^
		*A. ipsilon*	94.56 ± 1.49 ^a^	87.96 ± 3.13 ^a^	90.7 ± 9.47 ^a^
Pupal duration ^2^		*S. littoralis*	9.56 ± 1.23 ^c^	11.12 ± 1.68 ^b^	12.35 ± 1.79 ^a^
		*A. ipsilon*	16.85 ± 0.78 ^c^	18.09 ± 1.13 ^b^	19.55 ± 1.31 ^a^
Pupal weight	Male	*S. littoralis*	0.36 ± 0.03 ^a^	0.32 ± 0.06 ^b^	0.31 ± 0.03 ^b^
		*A. ipsilon*	0.37 ± 0.09 ^ab^	0.39 ± 0.07 ^a^	0.35 ± 0.03 ^b^
	Female	*S. littoralis*	0.42 ± 0.03 ^a^	0.34 ± 0.06 ^b^	0.33 ± 0.04 ^b^
		*A. ipsilon*	0.42 ± 0.09 ^a^	0.42 ± 0.07 ^a^	0.39 ± 0.06 ^a^
Emergency%		*S. littoralis*	100 ± 0 ^a^	100 ± 0 ^a^	93.33 ± 5.44 ^a^
		*A. ipsilon*	97.7 ± 3.25 ^a^	97.33 ± 3.77 ^a^	94.11 ± 8.32 ^a^
Fecundity	Female ^3^	*S. littoralis*	1263.33 ± 267.95 ^a^	1003.46 ± 291.08 ^a^	874.86 ± 171.09 ^a^
		*A. ipsilon*	393.26 ± 69.05 ^a^	375.26 ± 31.15 ^a^	312.73 ± 100.51 ^a^
Hatchability ^4^ %		*S. littoralis*	87.26 ± 2.96 ^a^	87 ± 2.11 ^a^	71.43 ± 3.49 ^b^
		*A. ipsilon*	88.23 ± 0.97 ^a^	80.53 ± 6.38 ^ab^	75.2 ± 3.16 ^b^

Means with the same letters do not significantly differ from each other (Tukey’s HSD or Dunn’s post-hoc test, *p* > 0.05). ^1^ Number of days from second-instar larvae to pupation; ^2^ Number of days from pupation to emergence; ^3^ Fecundity was estimated by counting the eggs from the first day through the sixth day (total number of eggs laid by one female); ^4^ Hatchability is calculated by counting the number of emerged larvae from collected eggs.

**Table 3:** Effect of exposure to LC_15_ and LC_50_ of cyantraniliprole via the leaf dipping method on sex ratio *of S. littoralis* and *A. ipsilon*.

	Sex	Control	Chi-Square (*p*-Value)	LC_15_	Chi-Square (*p*-Value)	LC_50_	Chi-Square (*p*-Value)
*S. littoralis*	Male	53.69 ± 2.43	0.55 (0.45)	59.26 ± 0.53	3.46 (0.06)	52.63 ± 8.57	0.28 (0.59)
	Female	46.23 ± 2.43		40.66 ± 0.53		47.3 ± 8.58	
*A. ipsilon*	Male	52.13 ± 1.86	0.18 (0.67)	49.27 ± 1.02	0.02 (0.88)	54.6 ± 5.73	0.84 (0.35)
	Female	47.87 ± 1.86		50.72 ± 1.02		45.39 ± 5.73	

**Table 4:** Effect of cyantraniliprole on *C. carnea* development.

Development	Application Method	Control	Concentrations		
			0.19 mg/L	0.37 mg/L	0.75 mg/L
Larval duration ^1^	Contact Method	9.32 ± 1.44 ^a^	9.11 ± 0.96 ^a^	9.28 ± 1.44 ^a^	9.71 ± 1.67 ^a^
	Feeding Method	9.18 ± 0.98 ^a^	8.82 ± 1.26 ^a^	9.14 ± 1.33 ^a^	9.55 ± 1.65 ^a^
Pupal duration ^2^	Contact Method	9.57 ± 1.34 ^a^	10.07 ± 2.74 ^a^	10.04 ± 2.61 ^a^	10.42 ± 2.64 ^a^
	Feeding Method	9.64 ± 1.62 ^a^	9.82 ± 2.53 ^a^	10.02 ± 2.79 ^a^	10.41 ± 2.85 ^a^
Pupation%	Contact Method	93.94 ± 7.48 ^a^	94.16 ± 7.27 ^a^	85.34 ± 15.1 ^a^	83.84 ± 10.05 ^a^
	Feeding Method	95.06 ± 6.14 ^a^	93.54 ± 8.81 ^a^	93.54 ± 8.81 ^a^	91.04 ± 8.39 ^a^
Emergence %	Contact Method	96.1 ± 5.57 ^a^	68.08 ± 18.95 ^ab^	56.66 ± 24.95 ^b^	53 ± 13.26 ^b^
	Feeding Method	93.46 ± 5.66 ^a^	74.66 ± 17.71 ^ab^	68.32 ± 18.56 ^ab^	58.31 ± 11.46 ^b^

After 24, 48, 72, en treatments was found between treatments Means with the same letter are not significantly different [Tukey’s HSD or Dunn post hos test, *p* > 0.05]; ^1^ number of days from second-instar larvae to pupation; ^2^ number of days from pupation to emergence.

## Data Availability

The datasets generated during the current study are available from the corresponding author upon reasonable request.
